# Development of a Sensing Platform Based on Hands-Free Interfaces for Controlling Electronic Devices

**DOI:** 10.3389/fnhum.2022.867377

**Published:** 2022-06-10

**Authors:** Mario Rojas, Pedro Ponce, Arturo Molina

**Affiliations:** Tecnologico de Monterrey, School of Engineering and Sciences, Mexico City, Mexico

**Keywords:** human-machine interface, speech control, head movements, eyes gestures, assistive technology, disabled people, multi platform

## Abstract

Hands-free interfaces are essential to people with limited mobility for interacting with biomedical or electronic devices. However, there are not enough sensing platforms that quickly tailor the interface to these users with disabilities. Thus, this article proposes to create a sensing platform that could be used by patients with mobility impairments to manipulate electronic devices, thereby their independence will be increased. Hence, a new sensing scheme is developed by using three hands-free signals as inputs: voice commands, head movements, and eye gestures. These signals are obtained by using non-invasive sensors: a microphone for the speech commands, an accelerometer to detect inertial head movements, and an infrared oculography to register eye gestures. These signals are processed and received as the user's commands by an output unit, which provides several communication ports for sending control signals to other devices. The interaction methods are intuitive and could extend boundaries for people with disabilities to manipulate local or remote digital systems. As a study case, two volunteers with severe disabilities used the sensing platform to steer a power wheelchair. Participants performed 15 common skills for wheelchair users and their capacities were evaluated according to a standard test. By using the head control they obtained 93.3 and 86.6%, respectively for volunteers A and B; meanwhile, by using the voice control they obtained 63.3 and 66.6%, respectively. These results show that the end-users achieved high performance by developing most of the skills by using the head movements interface. On the contrary, the users were not able to develop most of the skills by using voice control. These results showed valuable information for tailoring the sensing platform according to the end-user needs.

## 1. Introduction

The main purpose behind the concept of Electronic Aids for Daily Living, as presented by Little (2010), is to help persons with disabilities to operate electronic appliances. In addition, other similar concepts have appeared in recent years such as alternative computer access, environmental control systems, communication aids, and power mobility, which are closely related to the same idea of bringing more independence to individuals with diseases or physical limitations. As indicated in Cowan (2020), these systems allow people with disabilities to control devices in four categories: comfort (remote controls of temperature, windows, curtains, lights), communications (hands free telephone, augmentative, and alternative communication), home security (access to alarms or doors locks), and leisure (television or music appliances).

There have been studies to show positive aspects of using alternative control systems for users with disabilities, described as improvements in the quality of life, autonomy, and personal security (Rigby et al., 2011; Myburg et al., 2017). As a consequence, a lot of research has been done to develop hands-free and alternative input methods for Human Machine Interfaces (HMIs). For instance, Brose et al. (2010) presented emerging technologies for interacting with assistive systems, such as three dimensional joysticks, chin and head controls, computer vision to interpret face gestures from the user, voice recognition, control by eye gazes and head movements, Electro Encephalografic (EEG) signals, and Brain Computer Interfaces (BCI).

Commonly, the proposed interfaces are based on the remaining patient's abilities. For instance, head movement detection has been used as an input method. It was developed an application to activate phonetic systems for verbal communication in Chinese, which uses computer vision to capture features of head motion and distinguishes between nine gestures (Chang et al., 2020). A Human Computer Interface (HCI) for controlling a computer, based on head movements detected by a Microsoft Kinect was presented in Martins et al. (2015). Meanwhile, it was implemented the detection of facial expressions for navigating and executing basic commands in the computer, by using the Emotiv Epoc+ device (Šumak et al., 2019). Besides, an application was implemented by Lu and Zhou (2019) to manipulate the computer's cursor for typing and drawing, based on EMG signals for detecting five facial movements. Another alternative to implementing hands-free interfaces is based on eye tracking and gaze estimation. An algorithm to estimate the gaze point of the user's visual plane has been presented in Lee et al. (2021), which uses a head mounted camera and is described as less invasive and more comfortable for the user. Furthermore, a control based on Electro Oculography (EOG) signals for manipulating a quad copter in four directions: up, down, left, and right was implemented (Milanizadeh and Safaie, 2020); and EOG applications for activating smart home appliances have been developed (Akanto et al., 2020; Molleapaza-Huanaco et al., 2020). Meanwhile, several eye tracking techniques and related research with modern approaches such as machine learning (ML), Internet of Things (IoT), and cloud computing are summarized in Klaib et al. (2021); and a deep learning approach for gaze estimation is presented in Pathirana et al. (2022), which is indicated to perform robust detection in unconstrained environment settings. Finally, the voice commands have been used as an intuitive input method to control a system (Boucher et al., 2013). Recently, there have been developed new systems that incorporate techniques based on Artificial Neural Networks (ANNs) (Anh and Bien, 2021), which uses voice recognition to control a manipulator robot arm. In addition, it is presented in Loukatos et al. (2021) a voice interface to remotely trigger farming actions or query the values of process parameters.

In addition, multi modal schemes have been used to increment the number of possible instructions than those achievable with only one individual input. For instance, a multi modal HMI that incorporates EOG, EMG, and EEG signals was developed for controlling a robotic hand (Zhang et al., 2019). Besides, Papadakis Ktistakis and Bourbakis (2018) propose to use voice commands, body postures recognition, and pressure sensors to steer a robotic nurse. Again, in Sarkar et al. (2016) it was implemented a multi-modal scheme based on movements from the user's wrists and voice commands in order to manipulate the computer's cursor and execute the instructions. Also, EEG and EOG are combined for controlling a virtual keyboard for “eye typing” in Hosni et al. (2019).

On the other hand, there are long standing disorders that disconnect the person's brain from the body (paralysis), as a consequence, it is difficult to take advantage of the remaining abilities, such as voice commands or body movements. As a result, BCI is a technology developed for detecting the user's intentions to control an upper limb assistive robot, by using the P300 component obtained from EEG (Song et al., 2020). Similarly, a brain machine was developed by Zhang et al. (2017) to command a semi-autonomous Intelligent Robot for a drinking task. Hochberg et al. (2012) presented the feasibility to control an Arm Robot's actions obtained from neuron signals. Moreover, another study was developed by Fukuma et al. (2018) for decoding two types of hand movements (grasp and open), based on real-time Magnetoencephalography (MEG), to control a robotic hand. In addition, Woo et al. (2021) presented more applications of BCIs, such as those developed for controlling wheelchairs, computers, exoskeletons, drones, web browsers, cleaning robots, games, and simulators, among other systems.

Indeed, there have been important contributions these days, however, environmental control technology addresses other important issues: to fit the user's abilities, to be harmless, friendly and non-invasive, to be easy to maintain and low-cost, and to accomplish medical standards to be useful in real world environments. The control technology interface must meet the end-user's expectations and preferences in order to be successful, otherwise, the application will represent an obstacle and will be abandoned (Cowan et al., 2012). As an example, it is presented in Lovato and Piper (2019) that because of the availability of voice inputs in some electronic devices, children could search the internet when they are able to speak clearly, even before they have learned to read and write. Nowadays, devices like smart speakers (Amazon's Alexa, Apples'Siri) or microphone search services (Google, Youtube) have demonstrated that speech commands are a natural way to interact with computers. For those reasons, the community is calling for research in the areas of biomedical engineering to develop more attractive Human Machine Interfaces (HMI) to facilitate persons with disabilities the interaction with assistive devices or everyday home appliances. Expectation and evolution of interfaces are discussed in Karpov and Yusupov (2018), which remark other important aspects of present HMIs to be considered: intuitiveness, ergonomics, friendliness, reliability, efficacy, universality, and multimodality.

A traditional target for implementing and testing alternative interfaces has been the power wheelchairs. For that reason, Urdiales (2012) exposes different options to steer a wheelchair by using conventional joysticks, video game joysticks, tongue switches, touchscreens, early speech recognition modules, Electromyography (EMG), eye tracking based on Electrooculagraphic signals (EOG), and BCI. Another review of input technologies for steering a wheelchair is presented by Leaman and La (2017), where they summarized several methods based on biological signals, BCI, computer vision, game controllers, haptic feedback, touch, and voice. Despite the fact that there are several proposals for alternative inputs, only few of them have been evaluated with the end users to identify the real challenges and opportunities. According to the review paper presented by Bigras et al. (2020), the Wheelchair Skills Training Program (Dalhousie University, 2022) presents appropriate tasks for assessing power wheelchairs, the protocol is available and its scoring scales are well established. For that reason, 15 skills from the WST were adapted for the Robotics Wheelchair Test Skills (RWTS) by Boucher et al. (2013) to evaluate a smart wheelchair prototype. In this study, there were involved nine actively wheelchair users with years of driving experience. By using voice commands, the participants are able to request automated actions from the system in order to complete the tasks. Also, in Anwer et al. (2020) healthy users participated to evaluate an eye and voice controls for steering a wheelchair, however, only five skills tasks from the WST were used.

This article presents a modular system with different sensing methods for hands-free inputs. Moreover, the proposed platform includes hardware with several possibilities for connection to more electronic devices. Unlike other systems, this sensing platform is designed to be expanded for including new interfaces and adapted to different applications for environmental control systems. Thus, this platform has been tested in a power mobility application. The evaluation has been done by a standard protocol, involving volunteers with severe mobility problems and non-wheelchair users.

## 2. Materials and Methods

**Figure 1:**
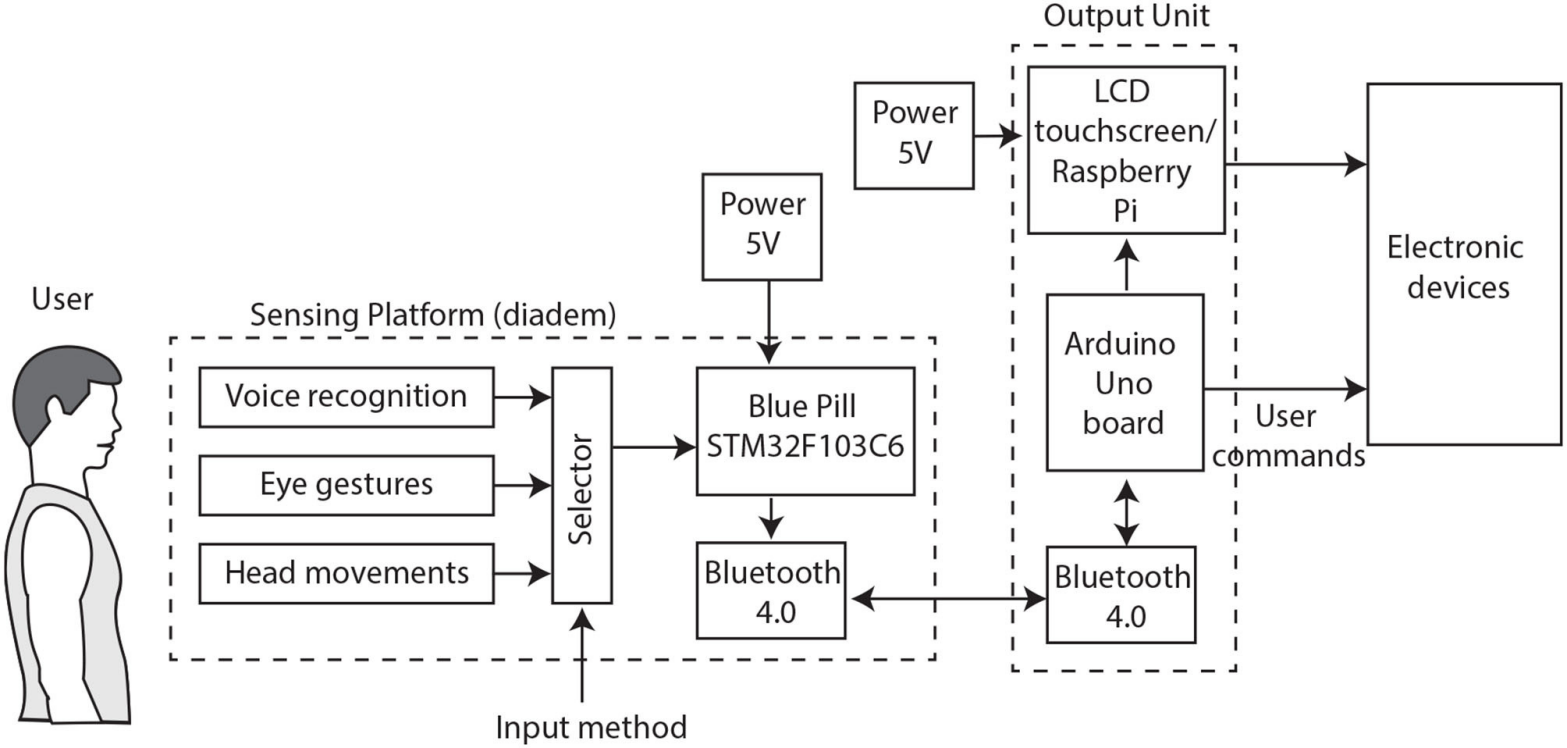
The block diagram illustrates the two main sections: the Sensing Platform or diadem and the Output Unit.

A general diagram of the system is illustrated in Figure 1, where two main blocks are indicated: the Sensing Platform (SP) and the Output Unit (OU). There are three input modes available in the SP: voice recognition, head movements, and eye gestures; which could be selected one at a time. A pair of Bluetooth modules, configured as “slave” and “master,” respectively; are used for sending data between the SP and the OU through serial communication. The OU processes the received data to identify the user's commands and generates control actions for electronic devices. The hardware and other components from both the SP and the OU are described in the next sections.

### 2.1. The Sensing Platform

The input modules are connected to a Blue Pill Development Board from STMicroelectronics, based on a 32-bit microcontroller STM32F103C6 (STMicroelectronics, 2022) that has an ARM Cortex-M3 core operating at a 72 MHz frequency. This microcontroller is programmed to acquire data from the selected input, process the information, and send it through the UART protocol. There are used Bluetooth 4.0 modules configured at 9,600 bauds per second for serial communication. These modules use low energy technology and high data transfers. In addition, a micro-USB connector is used to energize the Blue Pill, as shown in Figure 1. The 5V DC power is provided by a lightweight portable charger. This power bank has a capacity of 26,800 mAh with several standard ports used for power input and output.

**Figure 2:**
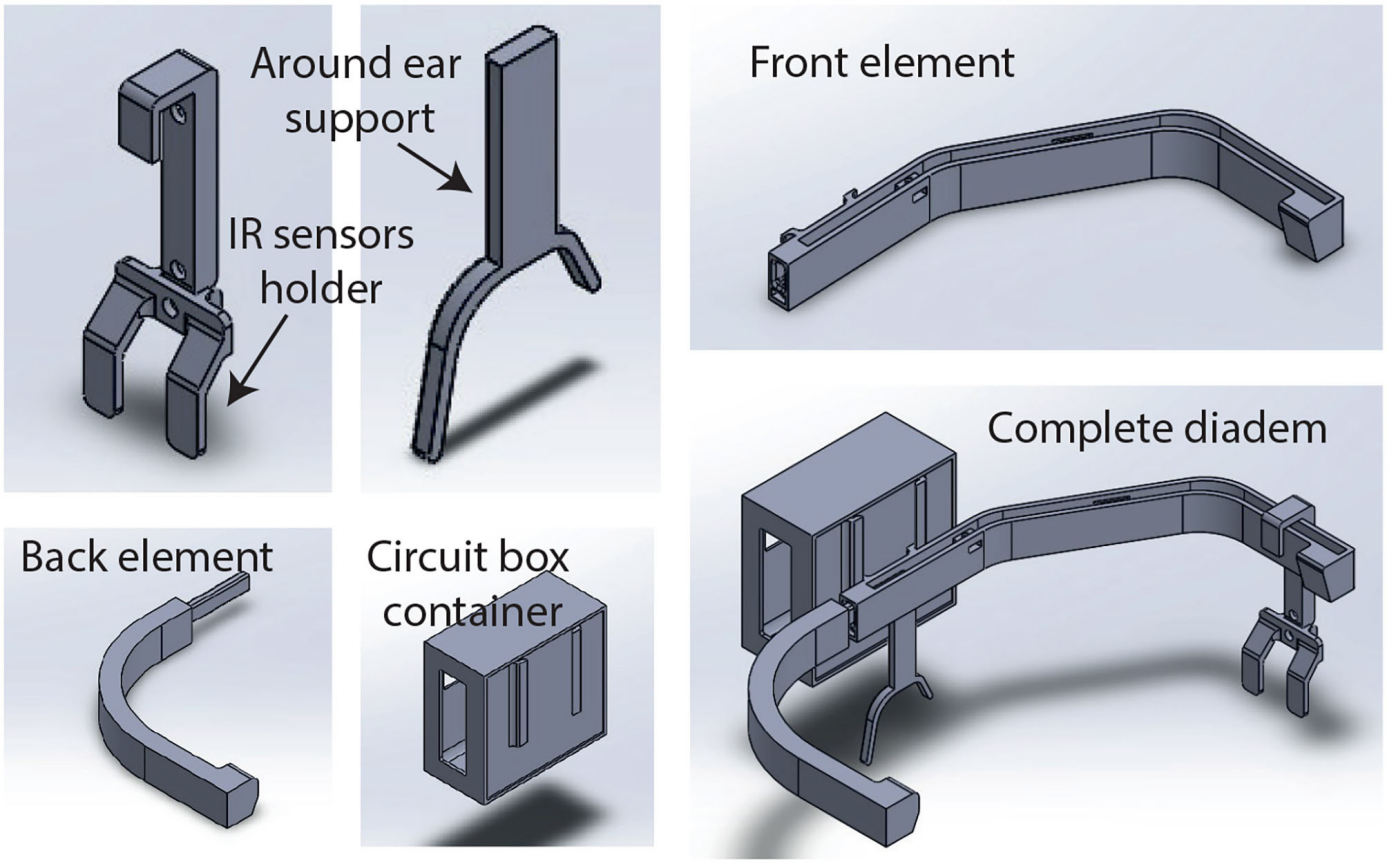
Three dimensional design of the diadem assembling pieces in SolidWorks.

A head-mounted wearable device (diadem) was designed to carry the electronic components for hands-free interfaces. A solidWorks model was created first, as illustrated by Figure 2. There are five parts for assembling the wearable device: the front and back pieces, the circuit box container, the ear rest piece, and the IR sensors holder. Under this scheme, it is possible to configure one interface without replacing the complete diadem. In the end, this design was printed with a solid material to be used during the prototype testing. The next sections describe the electronic modules used for the interfaces.

#### 2.1.1. The Voice Commands Module

**Figure 3:**
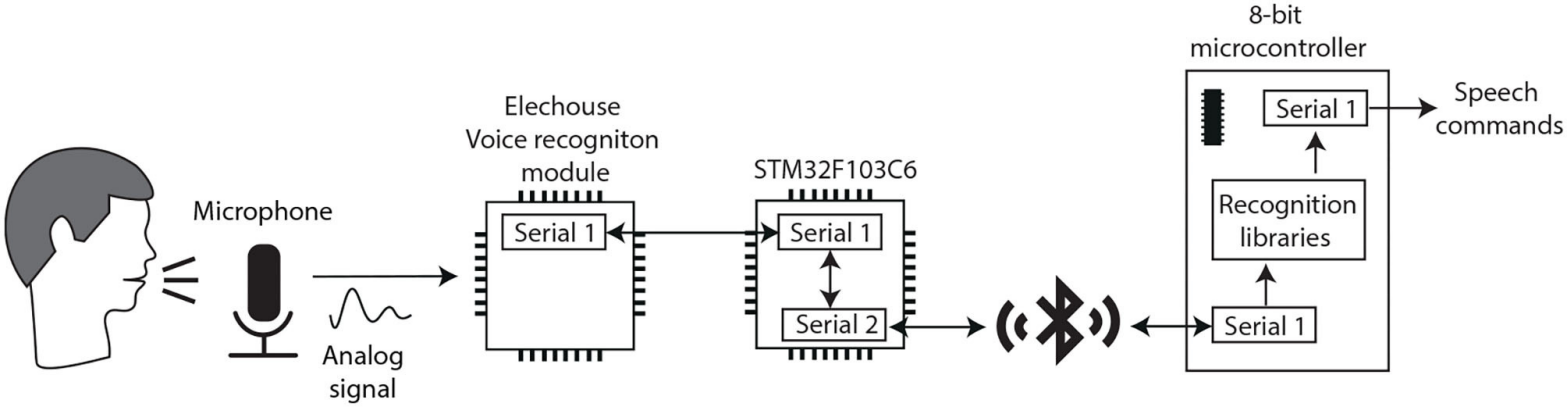
The block diagram of the voice recognition input interface.

For this first interface, an Elechouse Voice Recognition V3 module (Elechouse, 2022) is used, which is an integrated control speaking recognition board. This module is able to store 80 different voice commands and recognize seven at the same time, with a 99% accuracy under the ideal environment. It includes an analog input: a 3.5 mm connector for a mono channel microphone. Also, the small board has serial port pins for easy control and I2C pins. Before using it, the voice module needs to be configured with clear voice commands or even sounds, as there are users with limited speech or with difficulties speaking clearly. Moreover, there is no need to configure a specific language. The serial port control needs code libraries available at the Arduino UNO presented in Figure 1. For that reason, a serial communication path must be established, between the Elechouse board and the Arduino, through the Bluetooth modules and the STM32F103C6 ports as depicted in Figure 3.

#### 2.1.2. The Head Movements Interface

**Figure 4:**
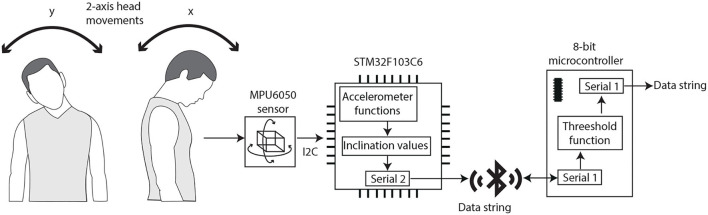
The block diagram of the head interface. There are shown the head movements of the user.

The second interface uses an MPU6050 sensor, which integrates a 3-axis accelerometer and gyroscope on the same board. The acceleration data, with a 16-bit resolution, is transmitted to the STM32F103C6 *via* the I2C protocol. The acceleration measurements are used to calculate the inclination angles when the users tilt their head in four directions: forward/backward, corresponding to the x-axis; and left/right, corresponding to the y-axis, as illustrated in Figure 4. To measure the inclination angles in both the x and y-axis, there were implemented in code the next mathematical relations:

$$\begin{array}{l} \theta_{x}=tan^{-1}\left(\frac{accel_{x}}{\sqrt{accel_{y}^{2}+accel_{z}^{2}}}\right)\left(\frac{180.0}{3.14}\right)\\ \theta_{y}=tan^{-1}\left(\frac{accel_{y}}{\sqrt{accel_{x}^{2}+accel_{z}^{2}}}\right)\left(\frac{180.0}{3.14}\right) \end{array}$$

Where θ_*x*_ and θ_*y*_ are the inclination angles running from −90 to 90°; and *accel*_*x*_, *accel*_*y*_, and *accel*_*z*_ are the acceleration values obtained from the sensor. Finally, a string with the movement angles is transmitted to the OU *via* the Bluetooth connection. In order to receive this data string, other devices like mobile telephones, tablets, or computers could be linked by a Bluetooth connection.

#### 2.1.3. The Eye Movements Interface

**Figure 5:**
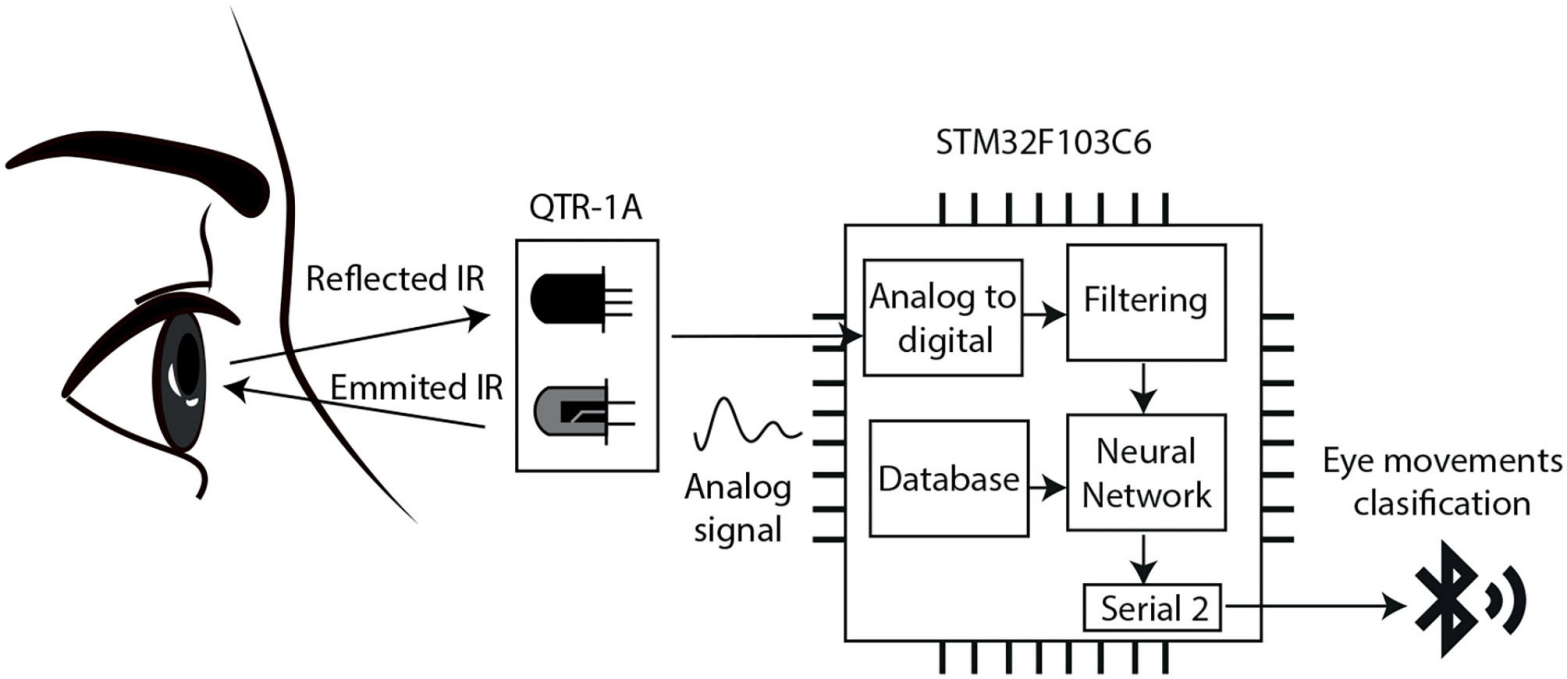
The block diagram of the eye gesture input based on Infrared Oculography.

Finally, the third interface is based on ocular movement detection for identifying eye gestures. The proposed circuit uses two reflectance sensors QTR-1A; which are small, lightweight, and carry an infrared LED and phototransistor pair. These sensors are typically used for edge detection and line following; however, for eye movement detection the emitter sends light to the eye, which is reflected from the frontal surface of the eyeball back to the phototransistor, as illustrated in the block diagram shown in Figure 5. The sensor produces an analog voltage output between 0 and 5 V as a function of the reflected light. This signal is received by two pins in the STM32FC103C6, configured as Analog to Digital Converter (ADC) inputs with 12 bits resolution. In the microcontroller, other code functions were deployed for smoothing the digitized signal and a DC blocker. In addition, it was implemented a special algorithm based on Artificial Neural Networks (ANNs) to determine the user's direction of the gaze and eye movements.

### 2.2. The Output Unit

The OU receives data from the SP, as presented in Figure 1. This information is processed by the Arduino ifbUNO board (Arduino, 2022), which incorporates an 8-bit microcontroller ATmega328P operating at 16 MHz. The implemented code in this processor includes libraries to easy-control the Voice Recognition module and functions developed to identify commands from the other interfaces. Also, there are included functions to generate outputs for controlling electronic devices. In addition, an LCD touchscreen is included in the OU, which is supported by a Raspberry Pi 4 Model B with Raspbian OS. The Raspberry Pi 4 was released with a 1.5 GHz 64-bit quad core ARM Cortex-A72 processor; connectivity through 802.11ac Wi-Fi, Gigabit Ethernet, and Bluetooth 5; besides 2 USB ports 3.0, 2 USB ports 2.0, 2 micro HDMI ports supporting 4 K@60 Hz. The touchscreen displays a Graphical User Interface (GUI) developed in the programming language Python. The GUI presents text strings from the information received by the Arduino UNO, as the readings from interfaces, the identified voice commands, and output values. Finally, it is necessary a 5V DC power supply for powering the Raspberry, however, this will be described in Section 3.1 as a part of the implementation study.

#### 2.2.1. Supported Communication Protocols to Electronic Devices

Several pins on the Arduino can be configured as basic communication peripherals: Universal Serial Asynchronous Receiver-Transmitter (UART), Inter-Integrated Circuit (I2C), Serial Peripheral Interface (SPI), and 14 general pins that can be used as digital input-output or as Pulse Width Modulation (PWM) outputs. Besides, the UART protocol allows the Arduino to communicate with computers *via* the USB port. On the other hand, the Raspberry Pi has a 40-pin header, with 26 General Purpose Input Output (GPIO) and power (3.3, 5 V) or ground pins. These GPIO pins enable the Raspberry Pi to communicate with other external devices in the real world. Furthermore, the GPIO set includes pins to be configured as well as simple inputs and output devices, other functions can be used: PWM, I2C, SPI, and UART.

#### 2.2.2. Network Communication

As there is growing technology for smart environments and interconnected devices relying on the Internet of Things (IoT), the Raspberry Pi acts as a “node” on a network to send and receive data. The “Python socket programming” enables programs to send and receive data at any time over the internet or a local network (Python, 2022). Python provides a socket class to implement sockets or objects for listening on a particular port at an IP address, while the other socket reaches out to form a connection, as a server and client scheme.

**Figure 6:**
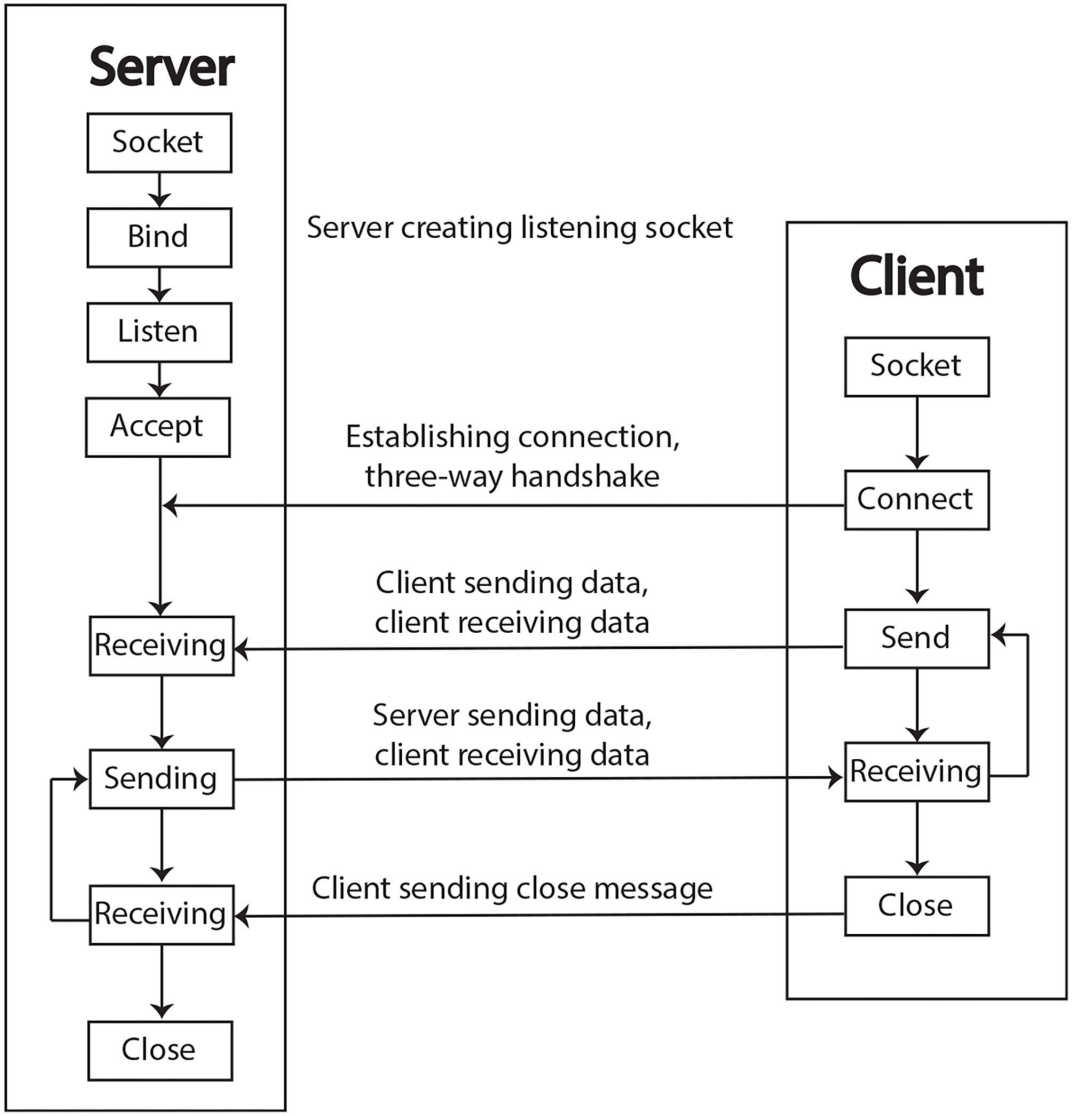
The block diagram of the socket flow connection taken from Python (2022).

The process to build socket objects in Python is described as follows: the first step is to import the socket library, then initialize an object with proper parameters, open a connection to an IP address with port or URL, send data, receive data, and finally close the connection. This process is depicted by the flow diagram in Figure 6. When creating a socket object, the default protocol is the Transmission Control Protocol (TCP) because of its reliability, as it ensures successful delivery of the data packets. Similarly, the Internet Protocol (IP) identifies every device across the internet and allows data to be sent from one device to another across the internet. Nowadays, the TCP/IP model is the default method of communication on the internet. As a consequence, networking and sockets expand the boundaries for developing remote control applications.

**Figure 7:**
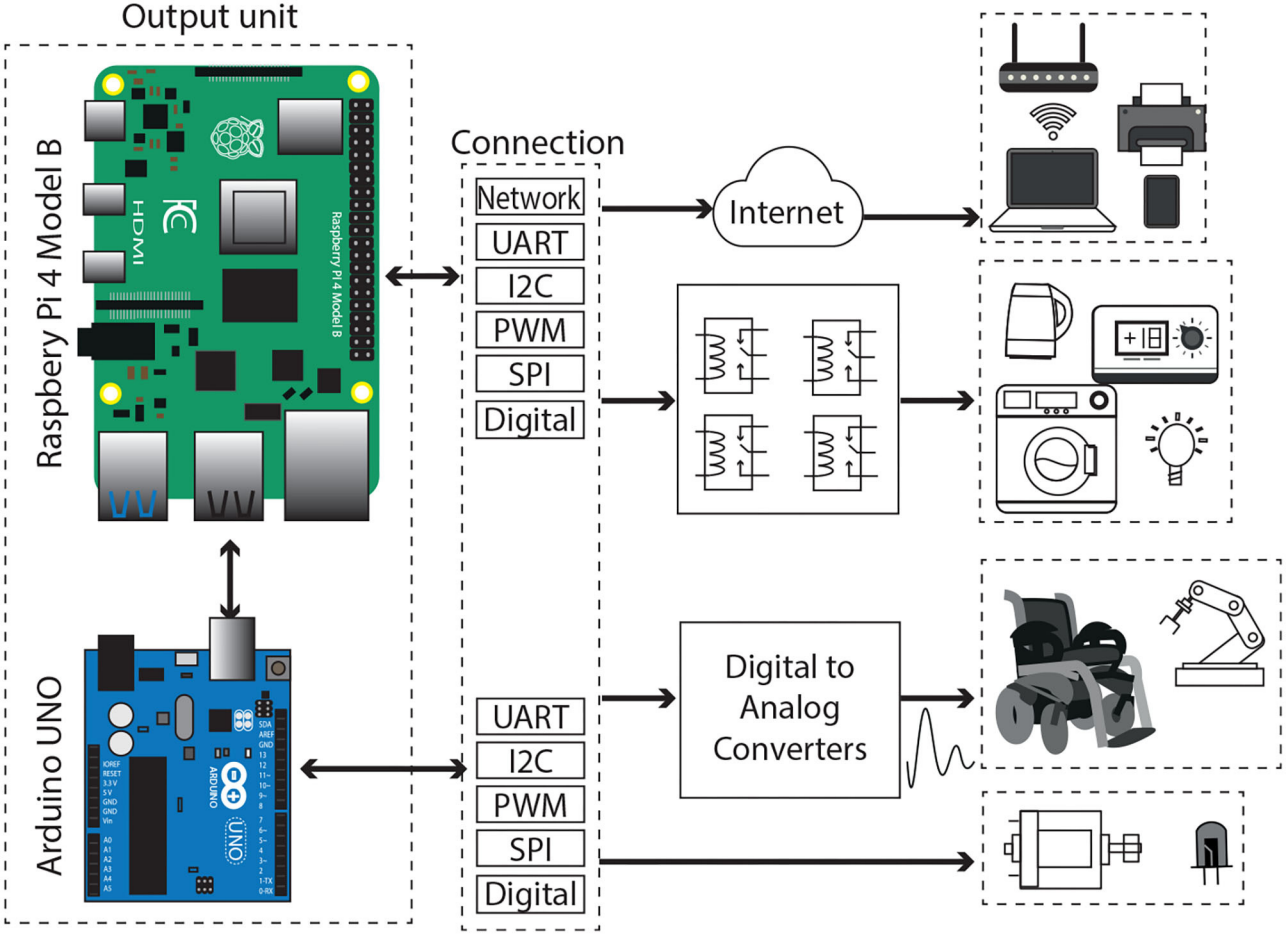
The block diagram of the Output Unit (OU) and its connectivity protocols.

In consequence, the OU possesses a large number of communication modalities for controlling smart home applications and networked devices, including recent trends on the Internet of Things (IoT). The output functionality is defined by the programmed code in the open-source Arduino Software (IDE) or the Raspberry libraries available for the Python IDE. The GPIO and the Arduino UNO peripherals are used by electric engineers for communicating to external devices such as computers, robots, electric appliances, house sensors, motors, alarms, lights, etc., as illustrated in Figure 7.

## 3. Results

### 3.1. An Application Case for Controlling a Power Wheelchair

Commercial models of power wheelchairs incorporate a conventional joystick for steering, however, there are people with severe disabilities who find it difficult to use it because of their physical limitations. Consequently, smart wheelchairs explore alternatives and technologies for interfaces, which are hands-free and non-invasive (Urdiales, 2012; Leaman and La, 2017). To address the issue, a commercial wheelchair was modified to incorporate the SP and the OU. This electric wheelchair is powered by two 24 V/20 Ah batteries, providing 8 h of autonomy under normal conditions, as indicated on the distributor website (Powercar, 2022).

**Figure 8:**
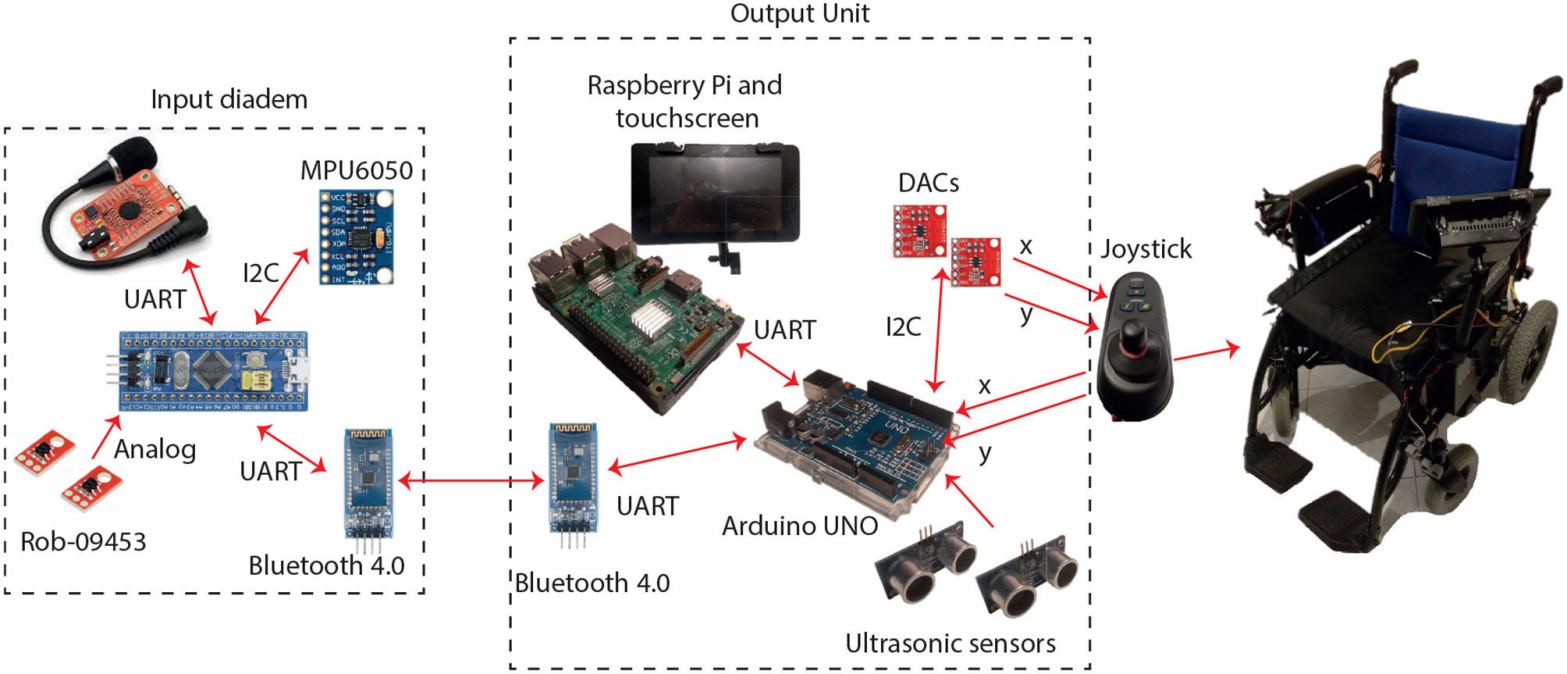
The power standard wheelchair and the components of the sensing platform and the Output Unit.

The default interface is a hand operated joystick, which integrates a 5 V DC USB-A output and is used to supply the Raspberry in the OU. Furthermore, the joystick provides two analog voltages, from 0 to 5 V, to control the wheelchair movements; however, they are substituted by outputs from two Digital-to-Analog Converters (DACs) MCP4725. These DACs use the I2C protocol for communicating with the Arduino. Also, an anti-collision module with ultrasonic sensors was installed in the wheelchair. Two sensors are placed in the front part of the wheelchair to detect close objects within a 1.2-m range. The installed hardware of the application and the communications protocols are shown in Figure 8.

**Figure 9:**
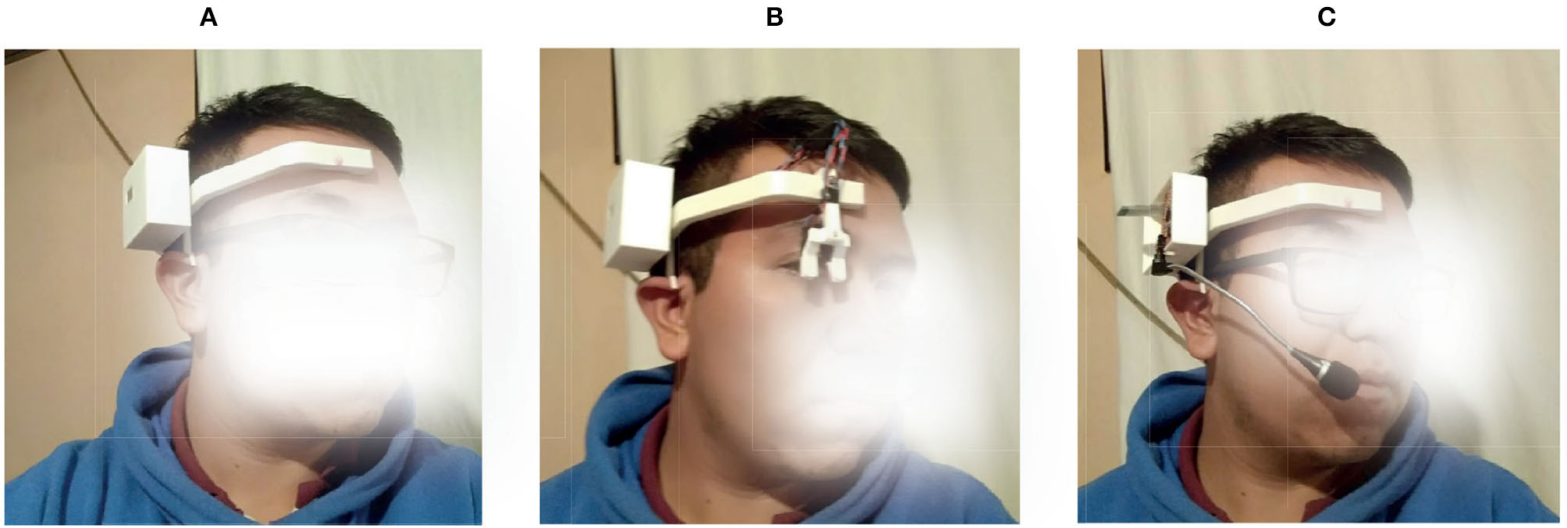
Different modules could be changed for the interface operation of the diadem. **(A)** Head interface, **(B)** Voice interface, **(C)** Eyes interface.

Figure 9 presents the printed diadem for the SP and its configuration for the hands-free interfaces. To successfully control the power wheelchair with the proposed system, several considerations were made as described next.

#### 3.1.1. Configuration of the Voice Commands Inputs

The speaking module was trained to recognize eight intuitive voice commands for controlling the wheelchair: “Stop,” “move,” “go,” “run,” “forward,” “backward,” “left,” and “right.” These commands are classified into “attention” and “orientation” categories, as the user might need continuous or discrete movements. To be more specific, an attention command has to be followed by an orientation command: e.g., say “move” +“right” to turn right for 2 s, or use “run”+“forward” for a continuous forward movement until another command is received. The commands are summarized and described in Table 1.

**Table 1:** Speech commands.

	**Command**	**Description**
Attention	Stop	Stop state/wait for an attention command
	Move	Configured to move during 1 s
	Go	Configured to move during 2 s
	Run	Configured to move continuously until another command is received
Orientation	Left	Turn to left direction
	Right	Turn to right direction
	Forward	Move to forward direction
	Backward	Move to backward direction

#### 3.1.2. Configuration of the Head Movements Interface

**Figure 10:**
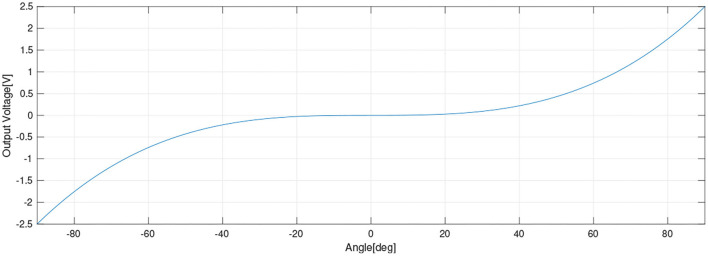
The cubic function to map the inclination angles to the wheelchair voltages.

Another alternative was to use the two-axis head movements to control the wheelchair; however, as the MPU6050 is very sensitive, slight head movements are detected as changes in the inclination angles and may cause wheelchair involuntary movements. For safety, a cubic function was implemented to map the inclination angles to the wheelchair operating voltages, as presented in Figure 10. The cubic function was chosen because it describes a quasi-linear zone in the middle of the curve; consequently, the wheelchair starts moving softly when the inclination angles reach the rising part of the curve and it remains still static in the middle “dead zone.” As a consequence, the users have to move their heads over a threshold angle to start moving the wheelchair. The mathematical relations for mapping the acceleration values to analog voltages are presented next.

$$\begin{array}{l} voltage_{x}=\frac{m{\left(accel_{x}-r\right)}^{3}}{s}+2.5\\ voltage_{y}=\frac{n{\left(accel_{y}-r\right)}^{3}}{s}+2.5 \end{array}$$

Where θ_*x*_ and θ_*y*_ are head inclination angles from −90 to 90°, *voltage*_*X*_ and *voltage*_*Y*_ are the control outputs voltages between 0 and 5 V, *m* and *n* are the slope factors for adjusting the flat zone of the function, and *r* is used for moving the quasi-linear zone along the x-axis, *s* is a scale factor and 2.5 is the reference voltage. By changing these values, the movement response could be adjusted according to the user's needs.

Finally, Table 2 is presented to summarize the operation of the head movements interface.

**Table 2:** The head movements configuration for controlling the wheelchair.

**Head movement**	**Action**
“Up”	Move forward
“Down”	Move backward
“Right”	Move to right
“Left”	Move to left

#### 3.1.3. Configuration of the Eye Gestures Interface

For configuring this recognition module, a virtual instrument was used to capture samples from both QTR-1A sensors during the four eye movements “open,” “close,” “left,” and “right.” The analog signal was sampled with a sampling rate of 50 Hz during a 1.5 s lapse. Another part of the acquisition process is filtering. After being digitized, the signal passes through digital filters to take out the noise and the DC component. First, it is used an Exponential Moving Average (EMA), which is the 1st infinite response filter for smoothing the signal without using much memory. This filter was implemented by using the recursive exponential relation presented next:

$$\begin{array}{l} y\left(n\right)=wx\left(n\right)+\left(1-w\right)y\left(n-1\right) \end{array}$$

where *x*(*n*) is the input value and *y*(*n*) is the output of the filter, at a moment in time *n*. Also, *y*(*n* − 1) is the previous output and *w* is the weighting factor.

Additionally, a DC blocker filter was used. The DC blocker is a 1st order Infinite Impulse Response (IIR) filter described by the next recursive equation:

$$\begin{array}{l} y\left(n\right)=\left[x\left(n\right)-x\left(n-1\right)\right]+\alpha y\left(n-1\right) \end{array}$$

where *x*(*n*) is the input value and *y*(*n*) is the output of the filter, at a moment in time *n*. Besides, *x*(*n* − 1) is the previous input value and *y*(*n* − 1) is the previous output value, and α determines the corner frequency.

**Figure 11:**
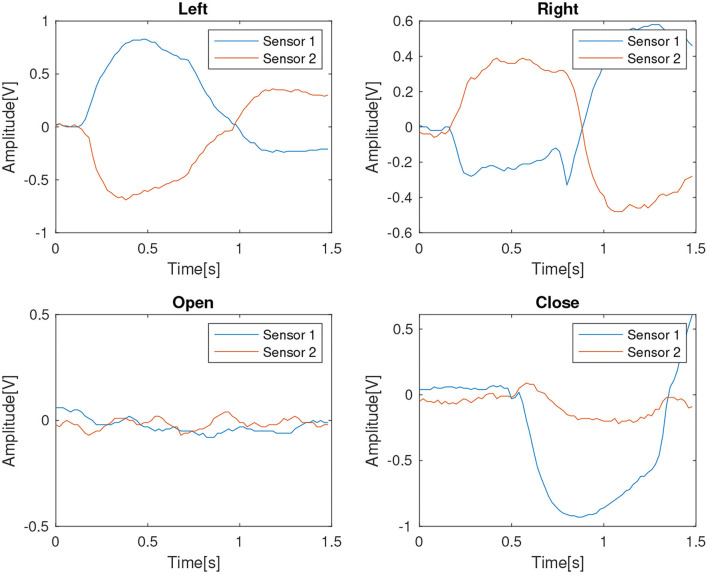
The block diagram of the eye gesture input based on Infrared Oculography.

To create the data base, the acquisition process was repeated 200 times to capture signals from the user making the left, right, open, and close eye gestures, 50 repetitions for each movement. In Figure 11, there are shown representative signals from the four eye gestures.

The collected data was saved as text files, then they were used as the training data base for the ANN. The implemented ANN uses a two-layer architecture with 10 neurons in the first layer and four neurons in the second layer. It used a heuristic approach to select this architecture, as there was needed a small topology that accomplishes well the classification requirements. The MATLAB Neural Network Pattern Recognition (Mathworks, 2022) was used to create and train the ANN. There were generated functions for both the ANN and the filters to be deployed on the Blue Pill. Finally, the embedded system uses a sequence of recognized eye gestures to control the wheelchair movements, as indicated in Table 3.

**Table 3:** The eye gesture configuration for controlling the wheelchair.

**Gestures sequence**	**Action**
“Left”,“Left”	Move to left
“Right”,“Right”	Move to right
“Right”, “Close”	Move forward
“Left”, “Close”	Move backward

### 3.2. Testing for the Application Case

#### 3.2.1. The Wheelchair Skills Test

The Wheelchair Skills Test (WST) (Dalhousie University, 2022) is a repeatable protocol for evaluating a well-defined set of tasks relevant to all wheelchair users. Expressly, 15 skills from the WST v. 4.3.1 were selected to be tested in the public space at Tecnológico de Monterrey Campus Ciudad de Mexico (ITESM-CCM) by using the head movements and the voice recognition interfaces. The selected skills are indicated in Table 4. The evaluation from the eye tracking module was not able to be completed, as there were important condition variations during the test because of condition changes in the different scenarios.

**Table 4:** Robotic WST capacities performance evaluation for volunteers A and B.

**ID**	**Skill**	**ID**	**Skill**
1	Rolls forward 10 m	9	Avoids moving obstacles
2	Rolls backward 5 m	10	Ascends small ramp
3	Turns 90° by moving forward	11	Descends small ramp
4	Turns 90° by moving backward	12	Ascends long curved ramp
5	Turns 180° in place	13	Descends long curved ramp
6	Maneuvers sideways	14	Rolls 2 m across side-slope
7	Gets through gate	15	Rolls 2 m on uneven surface
8	Rolls 100 m		

As described in the WST protocol, the scale for scoring the skills capacity is “2” (pass) if the task was completed safely and without difficulties, “1” (pass with difficulties) if the task was completed with collisions, excessive time or extra effort, and “0” (fail) is the task was aborted as it was considered unsafe. The Total Capacity is obtained by dividing the sum of the individual scores by twice the number of skills. For this case, the next equation was used.

$$\begin{array}{l} Total_{.}Capacity\left[\%\right]=\frac{\sum Individual_{.}Scores}{30}*100 \end{array}$$

#### 3.2.2. The Volunteers

Two volunteers with severe disabilities were recruited to validate the interfaces. Volunteer A is a 45-year-old male with spastic paraplegia because of a spinal cord injury and without his upper limbs, but he is able to speak and move his head without any problem. Volunteer B is a 19-years old male with quadriplegia, who is able to speak clearly but his head movements are a bit restricted because of damage to his spine. Both volunteers read an informed consent and authorized their participation. The trials were approved by the Ethics Committee of the Tecnológico de Monterrey, under registration number 13CI19039138.

#### 3.2.3. The Test

**Figure 12:**
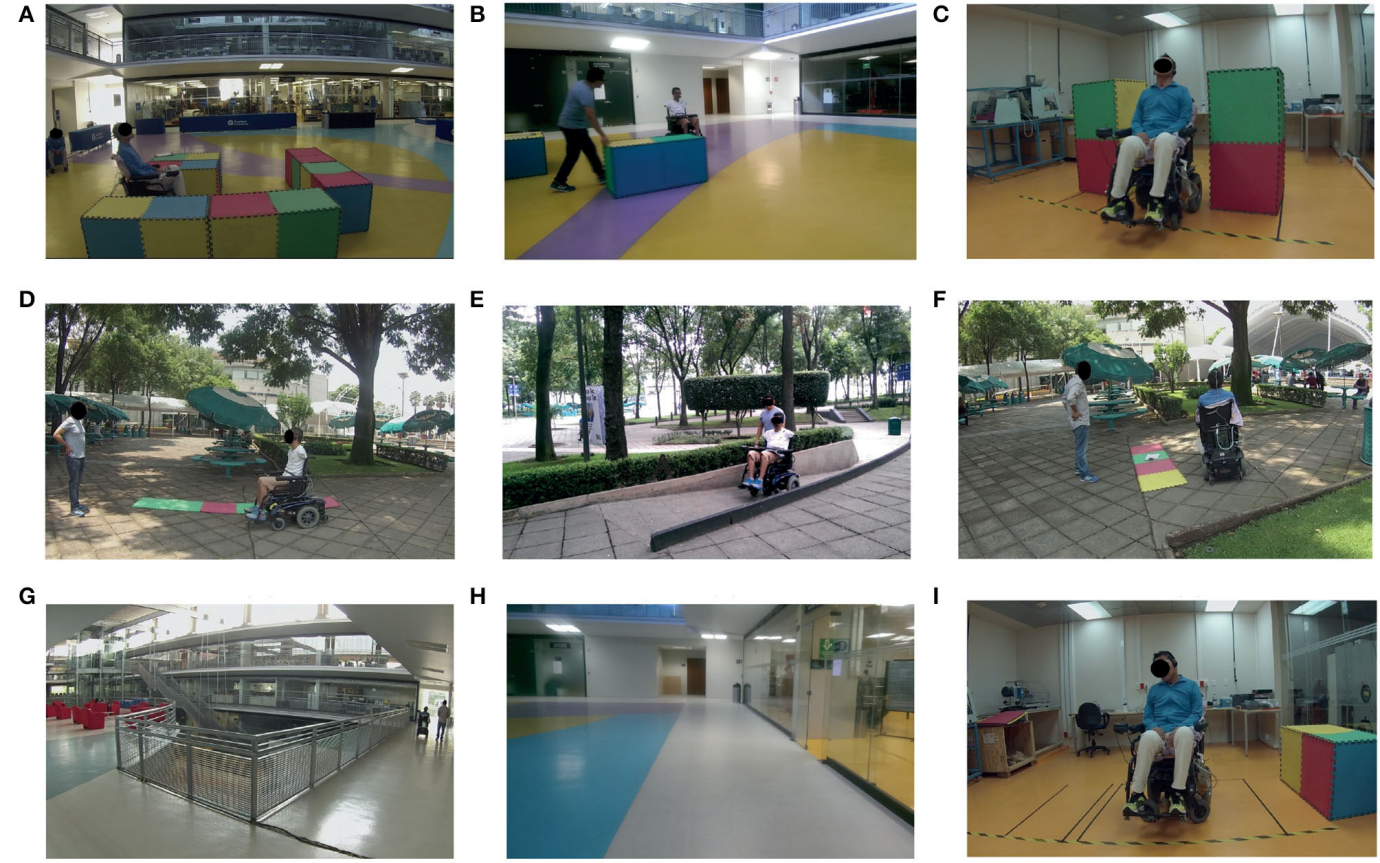
Available scenarios for the WST. **(A)** Turn 90°, **(C)** avoid obstacles, **(B)** gets through the door, **(D)** ascends/descends the small ramp, **(E)** ascends/descends curve ramp, **(F)** roll across side slope, **(G)** roll 100 m, **(H)** roll forward/backward, **(I)** turn 180°/maneuvers sideways.

The obstacles and scenarios described in the WST were adapted to the available conditions at the Campus, as presented in Figure 12. In addition, for safety issues, foam materials were employed to recreate some test scenarios like corridors and walls. During 2 days, 3-h sessions were planned for each participant to steer the wheelchair by using the SP. The first step was to familiarize with the SP, thus each volunteer experimented with the interfaces in an open space. After that, the participant was asked to complete the proposed 15 skills, in a single attempt.

#### 3.2.4. The Capacities Results

By using the head movements interface, both volunteers achieved most of the tasks without any problems. The Total Skills Capacity was 93.3% for volunteer A and 86.8% for volunteer B. On the other hand, by using the voice commands interface, both volunteers ranked similar results as they experimented with the same limitations to steering the wheelchair. The summarize the Total Skills Capacities, 63.3% was ranked for volunteer A and 66.6% for volunteer B. The individual scores are presented in Table 5.

**Table 5:** Wheelchair Skills Test capacities performance evaluation for volunteers A and B.

		**Volunteer A**		**Volunteer B**	
**ID**	**Skill**	**Head movements**	**Voice recognition**	**Head movements**	**Voice recognition**
1	Rolls forward 10 m	2	0	2	0
2	Rolls backward 5 m	2	0	2	0
3	Turns 90° by moving forward	2	2	1	2
4	Turns 90° by moving backward	1	0	2	0
5	Turns 180° in place	2	2	2	2
6	Maneuvers sideways	1	1	1	1
7	Gets through gate	2	2	2	2
8	Rolls 100 m	2	1	2	1
9	Avoids moving obstacles	2	1	2	2
10	Ascends small ramp	2	2	2	2
11	Descends small ramp	2	2	2	2
12	Ascends long curved ramp	2	2	2	2
13	Descends long curved ramp	2	2	2	2
14	Rolls 2 m across side-slope	2	0	0	0
15	Rolls 2 m on uneven surface	2	2	2	2
	**Total WST capacity [%]**	**93.3**	**63.3**	**86.6**	**66.6**

## 4. Discussion

Volunteers scored total capacities of 93.3 and 86.6% by using the head control; meanwhile, 63.3 and 66.6% were scored with the voice control. According to the results, both volunteers achieved well most of the skills by using head control. On the contrary, by using the voice module they experimented with problems to complete some tasks. The obtained scores for the head control test are closer to 100%; however, both volunteers experienced more problems commanding with the voice control. For comparison, the average capacity obtained by wheelchair users in Boucher et al. (2013) was 100 and 94.8%, by using a standard joystick and the vocal interface, respectively. The low scores shown in Table 5 indicate problems with controlling the wheelchair by voice commands; however, the volunteers achieved to complete most of the skills by themselves, not by using an automated system as indicated in Boucher et al. (2013). Indeed, automation is very helpful, but volunteers expressed satisfaction to be in charge and completing the task without any help from the system. In particular, tetraplegia has imposed a wide range of limitations and restrictions for Volunteer B, but he was able to increase their independent mobility using the SP. As indicated in Rigby et al. (2011), a key goal is to enable the patient's autonomy in activities that provide meaning purpose and enjoyment in their daily lives. The Volunteers expressed considerable expectations related to the use of alternative interfaces in their daily activities. Furthermore, the scores by using the head movements interface are promising and represent a high-level of control and independence for tetraplegics or persons with atrophied limbs. Volunteers agreed that the proposed input was easy to understand but training will be important for being more confident with the technology. Besides, it is important to consider parameter adjustments according to the user's needs, speed, and rhythm. In particular, volunteer A proposed modifications of the head movements interface as he felt to be more comfortable tilting down his head instead of upward, while Volunteer B preferred the opposite configuration.

The WST helped to collect valuable information about the interaction between users and interfaces; besides, opportunity areas for enhancing the proposed system. The WST is an important standard for training and assessing wheelchair skills (Bigras et al., 2020) with end users and specialized rehabilitation counselors to improve the sensing platform functionality. Although the scenarios were adapted to the Campus conditions, the test was challenging for the volunteers and the obtained scores contributed to growing positive evidence about the sensing platform for a practical application. This evaluation contributes to validating the end users' persistence to enhance their quality of life (Myburg et al., 2017).

### 4.1. Limitations

The voice control method presented problems because of the module's inability to recognize commands on time. Also, sometimes it is not easy for the user to speak accurate and clear vocal instructions while the wheelchair is moving, as they felt under pressure. Also, the voice control is limited to few instructions. In order to be more effective, it is important to train and configure more commands and implement new functions in the Output Unit. Meanwhile, for using the head as an input method, special training will be recommended in order to develop a more efficient control, however, this input method has proven to be very intuitive for the user. The eye tracking interface is still characterized in order to be evaluated by end users: ambient lighting, the emitter-detector response, and the continuous use of the infrared, as eyes tend to become dry and fatigued (Singh and Singh, 2012). It will be important to expand the testing protocol to the eye's gestures interface to collect more information from end users for better customization.

Despite the limited number of participants during the evaluation, target users were enrolled to validate the sensing platform with a power mobility application. In fact, both volunteers are non-wheelchair users, without experience to steer a power wheelchair and many difficulties to control one by conventional methods. Obviously, it was a challenging process to develop trials with unhealthy subjects, as they need several considerations to keep them safe and fewest work sessions than healthy subjects. Nevertheless, there are many advantages to including end users in the study, even if the sample is small. For this reason, the obtained results should be treated as guidance for future studies. Furthermore, there is a lack of testing and validation with end-users in similar proposals (Machangpa and Chingtham, 2018; Silva et al., 2018; Umchid et al., 2018).

Finally, the Raspberry Pi included in this design is limited to receiving and showing data on the touchscreen. Undoubtedly, there are further possibilities for developing connectivity to other applications but at this moment the voice module depends on libraries available at the Arduino. Therefore, a new version is being developed to embed all the systems in the Raspberry, including outputs for new applications.

### 4.2. Further Opportunities

As the proposed design is very modular, it is possible to transfer the implementation to another assistive technology such as a robotic arm or to control electronic devices in the user's environment. As indicated, customization is very important, however, it is fundamental to consider other conditions of the patient like mental capacities, logic, and verbal skills, emotions (tolerance, frustration), and physical conditions (force, head, and neck mobility). A key goal is to use the same platform for different applications, as the system is very modular. If the user feels fully accustomed to the sensing platform for steering his wheelchair, the next step is to adapt the same interface to command other devices like home appliances. Therefore, connectivity with other systems becomes a fundamental topic for hands free interfaces. Thus, connectivity with other services and systems is a fundamental topic in the development of technology. Further, there are new expectations for eye tracking applications (Trabulsi et al., 2021) and new trends, e.g., the use of Face Coding Action Systems, based on eye tracking, for emotions recognition (Clark et al., 2020).

More interfaces should be included in the sensing platform to tailor to other users. The proposed system supports patients with remaining abilities such as emitting vocal commands or sounds and controlling the head or eye movements. Nonetheless, if there are still remaining abilities in the patient for consciously executing basic tasks, there is an opportunity to adapt a sensing input for satisfying basic needs, like drinking water (Hochberg et al., 2012) or typing messages (Peters et al., 2020). As there are progressive diseases, it is a challenge to adapt systems to the patient's evolving needs. However, there are opportunities even for patients with disorders of consciousness, as interfaces could be designed to differentiate minimally conscious states from vegetative (Lech et al., 2019).

## 5. Conclusion

A modular system was presented for sensing multiple inputs to command a smart wheelchair. The implemented alternative inputs allowed users with strong disabilities to recover independence during the WST. Also, the tests with end users showed important feedback for customization and other opportunity areas for the system. Moreover, the employed hardware offers multiple possibilities for connectivity to other electronic devices and for exploring smart environments.

## Data Availability Statement

The original contributions presented in the study are included in the article/supplementary material, further inquiries can be directed to the corresponding author/s.

## Ethics Statement

The studies involving human participants were reviewed and approved by Ethics Committee of the Tecnológico de Monterrey under registration number 13CI19039138. The patients/participants provided their written informed consent to participate in this study. Written informed consent was obtained from the individual(s) for the publication of any potentially identifiable images or data included in this article.

## Author Contributions

MR, PP, and AM contributed with the idea of the smart wheelchair. MR and PP carried out the testing with volunteers. MR wrote the first draft of the manuscript. PP and AM wrote other sections of the manuscript. All authors reviewed the submitted version of the manuscript.

## Funding

This study was supported by a scholarship award from Tecnológico de Monterrey Campus Ciudad de México and a scholarship for living expenses from CONACYT.

## Conflict of Interest

The authors declare that the research was conducted in the absence of any commercial or financial relationships that could be construed as a potential conflict of interest.

## Publisher's Note

All claims expressed in this article are solely those of the authors and do not necessarily represent those of their affiliated organizations, or those of the publisher, the editors and the reviewers. Any product that may be evaluated in this article, or claim that may be made by its manufacturer, is not guaranteed or endorsed by the publisher.
